# Phenylalanine requirements using the direct amino acid oxidation technique, and the effects of dietary phenylalanine on food intake, gastric emptying, and macronutrient metabolism in adult cats

**DOI:** 10.1093/jas/skae009

**Published:** 2024-01-10

**Authors:** Jocelyn G Lambie, Júlia G Pezzali, Taylor L Richards, Jennifer L Ellis, Adronie Verbrugghe, Anna K Shoveller

**Affiliations:** Department of Animal Biosciences, Ontario Agricultural College, University of Guelph, Guelph, ON, CanadaN1G 2W1; Department of Animal Biosciences, Ontario Agricultural College, University of Guelph, Guelph, ON, CanadaN1G 2W1; Department of Grain Science and Industry, Kansas State University, Manhattan, KS 66506; Department of Animal Biosciences, Ontario Agricultural College, University of Guelph, Guelph, ON, CanadaN1G 2W1; Department of Animal Biosciences, Ontario Agricultural College, University of Guelph, Guelph, ON, CanadaN1G 2W1; Department of Clinical Studies, Ontario Veterinary College, University of Guelph, Guelph, ON, Canada N1G 2W1; Department of Animal Biosciences, Ontario Agricultural College, University of Guelph, Guelph, ON, CanadaN1G 2W1

**Keywords:** amino acid requirement, aromatic amino acids, carnivore, feline, satiety, stable isotopes

## Abstract

Despite Phe being an indispensable amino acid for cats, the minimum Phe requirement for adult cats has not been empirically defined. The objective of study 1 was to determine the minimum Phe requirement, where Tyr is in excess, in adult cats using the direct amino acid oxidation (**DAAO**) technique. Four adult male cats were used in an 8 × 4 Latin rectangle design. Cats were adapted to a basal diet for 7 d, top dressed with Phe to meet 140% of the adequate intake (NRC, 2006. Nutrient requirements of dogs and cats. Washington, DC: Natl. Acad. Press). Cats were randomly assigned to one of eight experimental Phe diets (0.29%, 0.34%, 0.39%, 0.44%, 0.54%, 0.64%, 0.74%, and 0.84% Phe in the diet on a dry matter [**DM**] basis). Following 1 d of diet adaptation, individual DAAO studies were performed. During each DAAO study, cats were placed into individual indirect calorimetry chambers, and 75% of the cat’s daily meal was divided into 13 equal meals supplied with a dose of L-[1-^13^C]-Phe. Oxidation of L-[1-^13^C]-Phe (F^13^CO_2_) during isotopic steady state was determined from the enrichment of ^13^CO_2_ in breath. Competing models were applied using the NLMIXED procedure in SAS to determine the effects of dietary Phe on ^13^CO_2_. The mean population minimum requirement for Phe was estimated at 0.32% DM and the upper 95% population confidence limit at 0.59% DM on an energy density of 4,200 kcal of metabolizable energy/kg DM calculated using the modified Atwater factors. In study 2, the effects of a bolus dose of Phe (44 mg kg^−1^ BW) on food intake, gastric emptying (**GE**), and macronutrient metabolism were assessed in a crossover design with 12 male cats. For food intake, cats were given Phe 15 min before 120% of their daily food was offered and food intake was measured. Treatment, day, and their interaction were evaluated using PROC GLIMMIX in SAS. Treatment did not affect any food intake parameters (*P* > 0.05). For GE and macronutrient metabolism, cats were placed into individual indirect calorimetry chambers, received the same bolus dose of Phe, and 15 min later received ^13^C-octanoic acid (5 mg kg^−1^ BW) on 50% of their daily food intake. Breath samples were collected to measure ^13^CO_2_. The effect of treatment was evaluated using PROC GLIMMIX in SAS. Treatment did not affect total GE (*P* > 0.05), but cats receiving Phe tended to delay time to peak enrichment (0.05 < *P* ≤ 0.10). Overall, Phe at a bolus dose of 44 mg kg^−1^ BW had no effect on food intake, GE, or macronutrient metabolism. Together, these results suggest that the bolus dose of Phe used may not be sufficient to elicit a GE response, but a study with a greater number of cats and greater food intake is warranted.

## Teaser

The minimum Phe requirement, when Tyr is provided in excess, was higher than the current recommendations indicating that more accurate recommendations for commercial adult cat food are necessary. The oral bolus dose of Phe used in this study was found to have no effect on food intake or macronutrient metabolism but tended to delay the time to peak gastric emptying (**GE**), suggesting potential functional properties that may influence the GE rate and play a role in promoting satiety.

## Introduction

The indispensable amino acid (**AA**) Phe is an aromatic AA that is used for protein synthesis and serves as a precursor to Tyr, a conditionally indispensable AA that can be further metabolized to produce catecholamines (Raper and Wormall, 1923; Hilton et al., 1998), the pigment melanin (Anderson et al., 2002), or thyroid hormones (Dratman., 1974). Prolonged (11 wk to 9 mo) restriction of dietary Phe in growing kittens resulted in neurological dysfunction, loss of black fur pigmentation, and weight loss (Rogers and Morris, 1979; Yu et al., 2001; Anderson et al., 2002; Dickinson et al., 2004). Currently, the only dose–response studies that empirically estimated Phe requirements were conducted using maximal growth (Anderson et al.,1980), nitrogen retention (Williams et al.,1987), and coat color (Anderson et al., 2002) of growing kittens as the dependent variables. From these three studies, when using purified diets in which AA is assumed to be 100% bioavailable, the minimal requirement (**MR**) for Phe + Tyr was 13.83 g·kg^−1^ with Tyr sparing roughly 50% of the Phe requirement (wt:wt). When these studies are considered together, the National Research Council’s *Nutrient requirements of dogs and cats* (NRC, 2006) defines the aromatic AA (Phe + Tyr) MR for growing kittens to sustain entirely black hair coats and maximum nitrogen retention at 15.3 g-Phe·kg^−1^ diet with a metabolizable energy value of 4.0 kcal·g^−1^. To address the lower bioavailability of Phe and Tyr among commercial diets, the National Research Council (**NRC**) recommended allowance (**RA**) for Phe in growing kittens is 5.0 g·kg^−1^ diet when the total AA is supplied at 19.1 g·kg^−1^ diet.

Although the adequate provision of dietary Phe is important to ensure the health of cats, to date, there are no published studies that have empirically determined the minimum Phe requirement in adult cats at maintenance. Consequently, the minimal Phe requirement for growing kittens determined from the studies has been extrapolated to provide estimates of adequate intake (**AI**) and RA for adult cats at maintenance (NRC, 2006). Extrapolating AA requirements from different life stages and from studies that applied the nitrogen balance technique, which is insensitive in mature animals, may lead to inaccurate recommendations. Estimates of AA requirements using the nitrogen balance technique are roughly 40% lower than those obtained through carbon oxidation techniques (Zello et al.,1995; Humayun et al., 2007; Elango et al., 2008, 2012). This was observed in dogs, where the MR estimations of AA were higher when assessed using carbon oxidation techniques (Shoveller et al., 2017; Templeman et al., 2019; Mansilla et al., 2020a,b; Sutherland et al., 2020) than when estimated with nitrogen balance or growth. Phenylalanine requirements were assessed in two different cohorts of dogs, one being greater (Shoveller et al. 2017) than and one being less than (Mansilla et al. 2018) the MR in NRC (2006). Thus, there is a need to determine the minimum Phe requirement in adult cats using these more sensitive and accurate techniques. The indicator amino acid oxidation (**IAAO**) technique has been recently applied in adult cats (Pezzali et al., 2023b) and a steady-state isotope dilution protocol for carbon oxidation studies has been determined (Pezzali et al., 2023c) to allow the successful application of the minimally invasive IAAO in the domestic cat to determine AA requirements or to assess AA bioavailability. In addition to the oxidation of the labeled AA in carbon oxidation studies as an indirect measure of protein synthesis, other secondary physiological outcomes can be considered when establishing AA requirements. While intakes of Phe or Tyr higher than the proposed NRC (2006) RA have been reported to support melanin deposition in hair (Watson et al., 2018), the potential role of Phe in promoting the release of cholecystokinin (**CCK**), and consequently GE has not been investigated to date.

Considered to be a satiety signal, CCK is a hormone that is released by specific enteroendocrine cells in the upper small intestine, commonly referred to as I-cells, when food reaches the duodenum (Wang et al., 2011). Protein and fat induce CCK release (Hopman et al., 1985), but also some specific AA can stimulate CCK, such as tryptophan and Phe (Gibbs et al., 1973; Hira et al., 2008; Wang et al., 2011; Daly et al., 2013) which then binds to receptors on the pyloric sphincter and is thought to delay GE (Wang et al., 2011). Research in humans (Liddle et al., 1986; Enç et al., 2001) and other species (Debas et al., 1975; Yamagishi and Debas, 1978; Raybould and Tache, 1988) suggests that dietary Phe will result in the release of CCK and delay gastric emptying (**GE**). Previous findings suggest GE to be a key mediator for hunger and satiety (Hunt, 1980; Di Lorenzo et al., 1988); thus, if CCK stimulation can delay GE, satiety may be induced and ultimately food intake reduced. Additionally, the speed at which GE occurs has a direct impact on macronutrient metabolism, as evidenced by studies conducted by Marathe et al. (2013) and Richards et al. (2021) where the rate of GE influenced postprandial glucose concentrations. For example, rapid GE can cause a rapid increase in postprandial glucose that surpasses the ability of insulin to efficiently move glucose into tissues, and potentially trigger lipogenesis. Ultimately, this can contribute to the accumulation of body fat and result in an elevated risk for obesity. Backus and Rogers (1996) reported that a bolus of Phe (44 mg kg^−1^ BW) resulted in an increase in plasma CCK in cats but did not evaluate GE. As such, we wanted to understand if dietary Phe will alter the rate of food intake and whether changes in GE rate underpinned these.

Two major studies were carried out using adult cats at maintenance. The objective of study 1 was to empirically determine the minimum Phe requirement in adult cats using the direct amino acid oxidation (**DAAO**) technique when Tyr is supplied in excess. We hypothesized that the mean Phe requirement is greater than the current recommendations (AI and RA) by the NRC (2006). The objective of study 2a was to evaluate the effects of an oral dose of Phe (44 mg kg^−1^ BW) on food intake when compared to an isonitrogenous dose of Ala (23.7 mg kg^−1^ BW). We hypothesized that Phe supplementation would decrease food intake. Lastly, the objective of study 2b was to evaluate the effect of Phe (44 mg kg^−1^ BW) on GE and macronutrient metabolism, when compared to Ala (23.7 mg kg^−1^ BW) using the ^13^C-OABT (^**13**^**C-OABT**). We hypothesized that Phe would delay GE as well as reduce respiratory quotient (**RQ**) and energy expenditure (**EE**).

## Materials and Methods

The University of Guelph Animal Care Committee approved all experimental procedures for study 1 (AUP #4640) and studies 2a and 2b (AUP #4680), which were in accordance with national, provincial, and institutional guidelines for the care and use of animals in research. The cats used in all studies were deemed healthy before the start of the study, based on medical history, a physical exam, serum biochemistry profile, and complete blood count.

## Animals and Housing

All cats used in studies 1, 2a, and 2b were neutered male domestic shorthairs (Marshall’s BioResources, Waverly, NY, USA), and were group-housed at the Ontario Agricultural College at the University of Guelph (Guelph, ON, Canada) in a free-living environment (7.1 m × 5.8 m). The cats were only kept in individual crates during feeding, which occurred once per day at ~0700 hours. A 12:12 (L:D)-h cycle was maintained with lights turning on at 0700 hours and turning off at 1900 hours. Temperature and humidity were maintained and monitored daily, averaging 23.6 °C and 32.4%, respectively. Environmental enrichment was provided as toys, scratching posts, hide boxes, perches, beds, and climbing apparatuses. The room was cleaned and sanitized once daily, while litter boxes were scooped, and litter was added twice per day as needed. Cats were socialized with familiar individuals 5 d/wk for 2 h daily. Socialization included the following activities: general health assessment, brushing, petting, and voluntary play. Cleaning and socialization were done at the same time each day to ensure the cats had a consistent routine throughout the duration of the studies.

## Study 1: The Phe Requirement

### Body composition determination

Prior to the study, dual-energy x-ray absorptiometry scans were performed by a trained person 24 h after the last meal to determine lean soft tissue mass (**LSTM**). Cats were sedated using intramuscular dexmedetomidine hydrochloride (Dexdomitor, Zoetis, Kirkland, QC, Canada; 0.5 mg/mL; 0.015 mg·kg^−1^). Total body mass, fat mass, LSTM, and bone mass were measured through duplicate scans on Small Animal Mode with the thin setting using a fan-beam dual-energy x-ray absorptiometry (Prodigy Advance GE Healthcare, Madison, WI, USA). Cats were positioned in dorsal recumbency with forelimbs extended cranially and repositioned as necessary between scans, which were completed in ~10 min. Estimates for LSTM were obtained from the system software (enCORE Version 16; GE Healthcare, Madison, WI, USA). Results from both scans were averaged. Sedation was reversed by 0.015 mg·kg^−1^ atipamezole (Antisedan, Zoetis, Kirkland, QC, Canada) after the completion of the second scan.

### Diets and study design

Four cats with a mean body weight (**BW**) of 5.40 ± 0.87 kg (± SD) and a mean age of 2.98 ± 0.47 yr (± SD) were used. The power calculation was based on a 0.05 probability of type I error using the flux of ^13^CO_2_ raw data from dogs from Templeman et al. (2019) (SAS, v. 9.4, SAS Institute Inc., Cary, NC).

Prior to the start of the study, the cats transitioned from a commercial diet (T22 Total Grain-Free, Nutram Pet Products, Elmira, ON; metabolizable energy = 3,835 kcal·kg^−1^; moisture = 10%, crude protein, min = 36%; crude fat, min = 19%; crude fiber, max = 5.5%) to the experimental basal diet over an 8 d period (transition period), where cats were fed to maintain BW based on historical colony feeding records. The experimental basal diet consisted of a semi-synthetic diet formulated to meet or exceed all nutrient requirements according to the NRC (2006), with the exception of Phe (Table 1). The experimental basal diet was prepared fresh daily as described by Pezzali et al. (2023a). During the transition period, the cats were offered 75% of their daily energy requirement as kibble and 25% as the experimental basal diet for 2 d, followed by 50% kibble and 50% experimental basal diet for 2 d, 25% kibble and 75% experimental basal diet for 2 d, and finally 100% experimental basal diet for the last 2 d of the transition period. Then, cats were offered a 100% basal diet (215.72 ± 4.83 kcal·d^−1^; mean ± SEM) for 7 d to adapt to the protein intake (adaptation period). Cats were fed twice daily (0800 and 1600 hours), with half of their daily meal offered at each feeding time. Food was available for 1 h during feeding times, and freshwater was provided ad libitum. Over the transition and adaption periods, a Phe solution (6.67 g·L^−1^ and no Ala) was added to the experimental basal diet to ensure the dietary provision of 140% of the NRC (2006) Phe recommendation for AI. Excess Tyr was provided to ensure Phe is shunted to protein synthesis or oxidation and not to Tyr production (Shiman and Gray, 1998).

**Table 1.** Ingredient composition of basal diet on as-fed basis and analyzed nutrient content on a dry matter basis of the basal diet in the direct amino acid oxidation study evaluating the minimum Phe requirement of adult cats (study 1)

Ingredient	g kg^−1^
Pregelatinized wheat starch^1^	287.00
Water	210.00
Poultry fat	135.00
Amino acid premix^2^	134.00
Lamb meal	85.00
Sucrose	50.00
Cellulose	23.00
Dicalcium phosphate	15.00
Sodium bicarbonate	15.00
Palatant^3^	13.00
Brewer’s yeast	10.00
Potassium chloride	8.00
Calcium carbonate	6.00
Choline	4.00
NaCl	3.00
Vitamin premix^4^	1.00
Mineral premix^5^	1.00

Chemical composition	Analyzed content
Dry matter (DM), %	76.77
Metabolizable energy^6^, kcal kg^−1^	3224
Crude Protein, % DM	22.81
Fat, % DM	20.26
Ash, % DM	8.96
Nitrogen-free extract, % DM	47.97
Amino acids, % DM	
Ala	1.60
Arg	1.79
Asp	0.85
Cys	0.98
Glu	3.75
Gly	1.80
His	0.35
Ile	0.84
Leu	1.69
Lys	1.15
Met	0.33
Met + Cys	1.31
Phe	0.29
Phe + Tyr	2.17
Pro	1.44
Ser	1.03
Thr	0.65
Trp	0.32
Tyr	1.87
Val	0.85

^1^PAYGEL 290 Pregelatinized Wheat Starch, Archer Daniels Midland Company, USA.

^2^Amino acid premix contained per kilogram: 26.00 g L-glutamic acid, 16.00 g L-tyrosine, 10.00 g L-arginine and L-leucine, 8.00 g L-alanine, glycine and L-cysteine, 7.00 g proline, 6.00 g L-lysine, L-threonine, and L-serine, 5.00 g L-isoleucine, 4.00 g L-valine, taurine, and L-asparagine H_2_O e, 2.00 g DL-methionine, L-tryptophan, and L-histidine HCl H_2_O.

^3^PALASURANCE C45-140 Dry, Kemin Nutrisurance, Inc., USA.

^4^Vitamin composition of the premix: 105.83 mg/kg biotin, 1,573 mg/kg folic acid, 91,700 mg/kg niacin, 7,020 mg/kg pantothenic acid, 6,350 mg/kg pyridoxine, 4,930 mg/kg riboflavin, 10,100 mg/kg thiamin, 24.94 mg vitamin B12, 13,079,000 IU/kg vitamin A, 412,000 IU/kg vitamin D3, 46,718 IU/kg vitamin E.

^5^Mineral composition of the premix: 1.55% calcium, 0.0348% phosphorus, 0.4658 magnesium, 13.92% potassium, 0.2% sodium, 12.93% chloride, 11.90% sulfur, 15,493.9 ppm copper, 92,272.1 ppm iron, 9,397.4 ppm manganese, 361 ppm selenium, 104,105.8 ppm zinc, and 1,953 ppm iodine.

^6^Metabolizable energy = 3.5 kcal/g × crude protein (%) + 8.5 kcal/g × acid-hydrolyzed fat (%) + 3.5 kcal/g × nitrogen-free extract (%); nitrogen free extract (%) = 100% − (crude protein % + acid-hydrolyzed fat % + crude fiber % + ash %).

After the adaptation period, the study was conducted as an 8 × 4 Latin rectangle design. We conducted the trial as an 8 × 4 Latin rectangle to better randomize the order of treatments, as opposed to a 4 × 4 incomplete Latin square design. All cats received all 8 diets in random order and no diet was duplicated within each DAAO study day. The cats were randomly assigned to one of the eight test diets and fed at 14 g·kg^−1^ BW. The test diets were prepared by supplementing the basal diet with one of eight L-Phe (99% L-Phe, Thera-Plantes Inc., Saint-Laurent, QC) solutions (0, 0.84, 1.69, 2.53, 4.22, 5.91, 7.60, and 9.28 g·L^−1^) at 6.30 mL·kg^−1^. To maintain similar nitrogen content among all solutions, L-Ala (≥99% L-Ala, Sigma-Aldrich, St. Louis, MO) was added to the solutions (5.01, 4.55, 4.10, 3.64, 2.73, 1.82, 0.91, and 0 g·L^−1^ for solutions 1 to 8, respectively). The final Phe content (% dry matter [**DM**] basis) in the test diets plus the dressing solution were 0.29%, 0.34%, 0.39%, 0.44%, 0.54%, 0.64%, 0.74%, and 0.84%. The study was carried out using a 2-d feeding regimen: 1) day 0: a 1-d adaptation period to the test diet, where the cats were fed 14 g·kg^−1^ BW (maintenance of BW); 2) day 1: the DAAO study and indirect calorimetry were conducted simultaneously. Following each DAAO study, cats were switched to a new test diet for 1 d, and the following day another DAAO study was conducted. This 2-d feeding regimen was repeated eight times until all cats consumed each test diet.

### DAAO studies

The feeding and isotope protocol for each DAAO study day was conducted according to Pezzali et al. (2023c,d). The total amount of food (75% of the animal’s daily food allowance; 10.5 g·kg^−1^), priming dose (0.44 mg·kg^−1^BW) of NaH^13^CO_3_ (99%; Cambridge Isotope Laboratories, Inc., Tewksbury, MA) and priming (4.80 mg·kg^−1^) and constant (1.04 mg·kg^−1^) doses of L-[1-^13^C]-Phe (99%; Cambridge Isotope Laboratories, Inc.) were based on the BW measured on the morning of each DAAO study day. The timeline for carbon oxidation studies in cats has been described in greater detail by Pezzali et al. (2023c).

## Sample Collection and Analysis

### Diet analysis

The AA content in the basal diet was analyzed by Ajinomoto Animal Nutrition North America, Inc. (Chicago, IL) (total AA [AOAC 994.12], Tryptophan [IAO 13904:2005 E]). The basal diet was also analyzed for moisture (AOAC 930.15), crude protein (AOAC 990.03), hydrolyzed fat (AOAC 945.16), and ash (AOAC 942.05) at a commercial laboratory (SGS Agri-food Laboratories, Guelph, ON, Canada).

### Breath samples and calorimetry data

Calorimetry data were collected automatically using Qubit calorimetry software (Customized Gas Exchange System and Software for Animal Respirometry; Qubit Systems Inc., Kingston, ON, Canada). The flow rate was measured with a mass flow meter (#F250, Qubit Systems Inc.). Concentrations of carbon dioxide (CO_2_) and oxygen (O_2_) gases within the chambers were measured via CO_2_ (QS151, Qubit Systems Inc.) and O_2_ (Q-S102, Qubit Systems Inc.) analyzers. Measured volume of CO_2_ (**VCO**_**2**_ during fasting and fed states) were averaged over the collection periods to obtain mean fasting and fed VCO_2_ for each cat. Background and enriched samples of CO_2_ were collected by trapping subsamples of expired CO_2_ in 8 mL of 1 M NaOH. Samples were transferred to serum collection tubes (Vacutainer, Becton Dickinson, Franklin Lakes, NJ, USA; #366430), evacuated, and stored at −20 °C until further analysis. Enrichment of ^13^C in breath samples was performed using a Gasbench II interfaced with a Delta V Plus mass spectrometer (Thermo Scientific, Bremen, Germany). Enrichment of CO_2_ samples was expressed above background samples as atom percent excess (**APE**).

### Calculations

The rate of ^13^CO_2_ released from Phe oxidation per kilogram of BW (F^13^CO_2_, mmol·kg^−1^·h^−1^) was calculated as follows:


$$\begin{array}{l} F^{13}CO_{2\;}(mmol\cdot kg^{-1}\cdot h^{-1})=\frac{\left(VCO_{2}\times APE_{CO_{2}}\times 44.6\times 60\right)}{\left(BW\times 100\times 1\right)} \end{array}$$

where VCO_2_ is the average production of CO_2_ during fasting state (mL·min^−1^), $APE_{CO_{2}}$ represents the APE of ^13^CO_2_ during isotopic steady state (%), BW is the cat’s BW expressed as total BW (kg) or LSTM (kg), 44.6 (mmol·mL^−1^) and 60 (min·h^−1^) convert the VCO_2_ to micromoles per hour, the factor 100 changes APE to a fraction, and 1.0 is the retention factor of CO_2_ due to bicarbonate fixation based on previous dog report (Shoveller et al., 2017). Fasting VCO_2_ was used because of the increased variability associated with fed-state VCO_2_ in free-living animals.

Resting energy expenditure (**REE**) and fed-state energy expenditure (**FEE**) were calculated based on the abbreviated Weir equation (Weir, 1949):


$$\begin{array}{l} EE\;(kcal)=3.94\times O_{2}\;consumed\;(L)+1.11\times CO_{2}\;produced\;(L) \end{array}$$

The EE was further expressed in relation to BW and metabolic BW (BW^0.67^) for all cats. The Qubit system software calculates the RQ based on the ratio of CO_2_ produced and O_2_ consumed.

### Statistical analysis

The study was designed and conducted as an 8 × 4 Latin rectangle design. The effect of Phe content in the test diet on F^13^CO_2_ was analyzed using PROC GLIMMIX of SAS (v. 9.4; SAS Institute Inc.) with diet as a fixed effect and cat as a random effect. The effect of dietary Phe content on F^13^CO_2_ was also fitted using competing statistical models, namely broken-line linear and broken-line quadratic, using the NLMIXED procedure of SAS where diet was treated as a fixed effect and cat as a random effect. The estimated mean breakpoint parameter and its corresponding 95% confidence interval were determined in each model. Models were then compared based on the Bayesian Information Criterion, where the lowest value represents the best fit.

## Study 2: Measurement of Food Intake (Study 2a), GE Rate and Macronutrient Metabolism (Study 2b)

### Study design

For studies 2a and 2b, 12 neutered male domestic shorthair cats with a mean BW (±SD) of 5.21 kg (±0.78) and a mean age (± SD) of 2.83 yr (± 0.39) were used. A sample size calculation for the outcome variable of gastrointestinal transit time was performed to determine the necessary number of cats required for this study using a power of 0.8, an α of 0.05, an effect size of 1.87, and SD of 4.93 using G*Power. This sample size calculation was based on a previous study investigating GE using isotopic markers in cats (Peachey et al., 2000) and indicated a sample size of six cats per treatment would allow for adequate statistical power to maintain a β equal to or greater than 0.80.

In both experiments, cats were randomly allocated into one of two groups (G1, *n* = 6; G2, *n* = 6). Each group was randomly assigned to treatment (TRT) or control (CTRL). Cats assigned to TRT received a dose of Phe (44 mg kg^−1^ BW) mixed with a salmon-flavored lickable cat treats (83 mg kg^−1^ BW) (Catit Creamy Lickable Cat Treat, Rolf C. Hagen Inc.), while cats on CTRL received an isonitrogenous dose of Ala (23.7 mg kg^−1^ BW) also mixed into the lickable treat (83 mg kg^−1^ BW) to ensure the equal molar provision of nitrogen, as protein has satiety-inducing effects. Cats were weighed the day before the experimental period (day −1) for food intake measurement, and GE rate, and macronutrient metabolism assessment to determine their respective dose of TRT or CTRL.

### Study 2a: food intake measurement

Study 2a was conducted as a crossover experiment with two periods of 4 d (days 0 to 3). Cats were fed (0700 hours) TRT or CTRL at time = 0 min, and at time = 15 min, the cats were then offered 120% of their daily ration of the commercial diet (T22 Total Grain-Free, Nutram Pet Products) to mimic owner feeding practices. Their daily ration of food was determined based on historical feeding records to maintain BW. The remaining food was periodically weighed to calculate the food intake rate and returned to the cats until all food was eaten. All uneaten food at time 30 min post-feeding was weighed and discarded to measure total food intake. After a 4-d washout period, cats were assigned to the other treatment and a second 4-d experimental period occurred as described above.

### Study 2b: Measurement of GE rate and macronutrient metabolism

For study 2b, GE rate and macronutrient metabolism were assessed in a crossover experiment with two periods of 5 d (days 0 to 4) in study 2b. Prior to study 2b, a pilot study was carried out with four neutered male domestic shorthair cats, with a mean BW (±SD) of 5.59 kg (±1.23) and a mean age (± SD) of 2.79 (±0.45) yr, to determine an efficient and accurate sampling technique to measure GE using the ^13^C-OABT. After 24 h of fasting, cats were weighed and placed into individual respiration calorimetry chambers to collect breath samples for GE analysis. GE was assessed using an isotope dilution technique via breath ^13^CO_2_ enrichment after ingesting a bolus dose (5 mg·kg^−1^ BW) of [1-^13^C]-octanoic acid. Once in the chamber and after 30 min of gas equilibration, three fasting respiration/indirect calorimetry measurements and breath samples were taken over three consecutive 20 min periods to determine the resting VCO_2_ and volume of O_2_ (**VO**_**2**_), and background enrichment of ^13^C. At time = 0 min, [1-^13^C]-octanoic acid (99%; Cambridge Isotope Laboratories, Inc.; 5 mg kg^−1^ BW) was added to 50% of their daily metabolizable energy requirement (T22 Total Grain-Free, Nutram Pet Products) and offered to the cat. This meal was consumed within 5 min by all four cats. Expired CO_2_ was collected every 20 min for the first 2 h and every 40 min for the next 10 h. Either all (22 breath samples; one breath sample every 20 min from 0 to 2 h and one breath sample every 40 min from 2 to 12 h) or half (11 breath samples; one breath sample every 40 min from 0 to 2 h and every 80 min from 2 to 12 h) of the breath samples collected were used for calculations and then compared to enable an efficient collection approach. Calorimetry data collection (VCO_2_ expired and VO_2_ consumed) occurred in 4 min periods, every 20 min, and the data over the period were averaged and used for GE analysis. Calorimetry data were collected using Qubit calorimetry software as described previously. Cats spent a total of 12 h within the chambers and were previously adapted to remain in the chambers for 24 h (Hogan et al., 2022).

With the protocol developed from our small pilot trial, study 2b consisted of 5 d (days 0 to 4). During days 0 to 3, cats were fed (0700 hours) their respective dose of TRT or CTRL, and 15 min after treatment consumption, they were fed their daily ration (100%) in amounts to maintain BW based on historical feeding records (T22 Total Grain-Free, Nutram Pet Products). On day 4, after 24 h of fasting, cats were weighed and placed into respiration calorimetry chambers to assess GE and macronutrient metabolism. Cats were then fed (time = 0 min) their corresponding dose of TRT or CTRL. At time = 16 min, [1-^13^C]-octanoic acid (99%; Cambridge Isotope Laboratories, Inc.; 5 mg kg^−1^ BW) was topically added to 50% of their daily ration and offered to the cat. This meal was usually consumed within 5 min. GE was assessed using half of the breath samples collected as validated in the pilot trial (11 breath samples; one breath sample every 40 min from 0 to 2 h and every 80 min from 2 to 12 h). Samples were collected as described above for both the collection of ^13^CO_2_ and determination of VCO_2_ and VO_2_. Cats stayed in the chamber for an additional 10 h following the completion of breath sample collection, for a total of 22 h of indirect calorimetry. Each calorimetry session consisted of a 1 h fasted period (−60 to 0 min), then the cats received their meal (0 min), followed by a 2-h fed-state (0 to 120 min) and three postprandial periods (6 h each) (120 to 480 min, 480 to 840 min, and 840 to 1,200 min) with the total postprandial state totaling 18 h (120 to 1,200 min) of readings following feeding.

### Sample collection, analysis, and calculations

Breath samples (background and enriched samples of CO_2_) were collected and stored frozen at −20 °C and analyzed for enrichment of ^13^C as described previously. Enrichment of CO_2_ samples was expressed as APE. The rate of F^13^CO_2_ expired (mmol min^−1^) was calculated using the following equation adapted from Peachey et al. (2000) who previously used the ^13^C-OABT to determine GE rate in adult cats:


$$\begin{array}{l} F^{13}CO_{2\;\;\;}(mmol\;\min^{-1})=\frac{\left(VCO_{2}\times APE_{CO_{2}}\times 44.6\right)}{100} \end{array}$$

The above formula for F^13^CO_2_ (mmol min^−1^) is not adjusted to BW as in study 1. This adjustment was intentionally omitted to facilitate comparisons with previous research on GE in cats using the ^13^C-OABT where BW correction was not applied (Peachey et al., 2000). These authors also calculated the rate of ^13^CO_2_ expired per hour, not minute, which explains the absence of a conversion of VCO_2_ from mmol min^−1^ to mmol h^−1^.

Calculated VCO_2_ during fasting, fed, and postprandial states were collected using Qubit calorimetry software and averaged to obtain mean fasting, fed, and postprandial VCO_2_ for each cat. Breath samples (background and enriched samples of CO_2_) were collected and stored frozen at −20 °C and analyzed for enrichment of ^13^C as described previously. The EE was calculated using the modified Weir equation (Weir, 1949). The production of ^13^CO_2_ (mmol min^−1^) was calculated as done in the pilot trial.

### Statistical analysis

Food intake rate, time to finish, and orts data were analyzed using a mixed model via PROC GLIMMIX of SAS (v. 9.4; SAS Institute Inc.), where treatment, day, and their interaction were treated as fixed effects, and cat and period were treated as random effects. Day was treated as a repeated measure within each period. Assumptions about residual normality from the model were assessed through visual inspection and using the Shapiro–Wilk test. A Tukey’s honestly significant difference test was used to separate significant lsmeans.

For the pilot trial, total GE, as assessed by area under the curve (**AUC**) of ^13^CO_2_ (mmol min^−1^) plotted against time, and time to peak ^13^CO_2_ production were analyzed using PROC GLIMMIX in SAS. Treatment (all vs. half) was treated as a fixed effect, and the cat was treated as a random effect. In study 2b, total GE was analyzed for AUC and time to peak using a mixed model via PROC GLIMMIX of SAS, where treatment was treated as a fixed effect, and cat and period were treated as random effects. Assumptions of model residual normality were assessed through visual inspection of residuals and using the Shapiro–Wilk test. A Tukey’s test was used to separate lsmeans when the main effect was significant. Fasted (−60 to 0 min), fed (0 to 120 min), postprandial 1 (120 to 480 min), postprandial 2 (480 to 840 min), postprandial 3 (840 to 1,200 min), and postprandial total (120 to 1,200 min) RQ and EE data were analyzed with PROC GLIMMIX of SAS, using the same aforementioned model. Data are reported as LSM ± SEM. Significance was declared when *P* < 0.05 and a trend when 0.10 < *P* ≤ 0.05.

## Results

### Study 1: the Phe requirement

**Figure 1.**
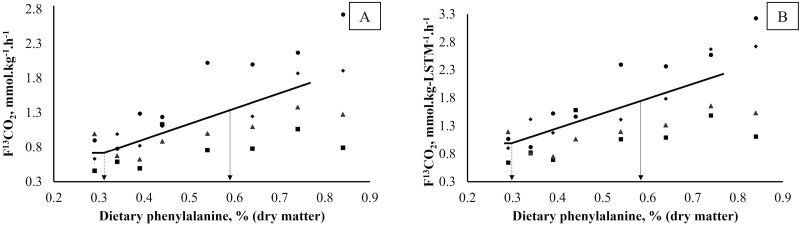
Influence of dietary Phe on production of ^13^CO_2_ from Phe oxidation (F^13^CO_2_) on adult cats using the DAAO technique. The F^13^CO_2_ is presented as a function of total body weight (A) and lean soft tissue mass (B). The breakpoint (dashed arrows) represents the estimated mean Phenylalanine requirement when Tyr is supplied in excess (0.32% in A and 0.31% in B). Solid arrows represent the upper 95% CL for Phenylalanine requirement (0.59% in A and 0.58% in B). A sample size of *n* = 4 was achieved for all diets.

No effect (*P* > 0.05) of dietary Phe intake was observed on BW, EE (fed and fasted), and RQ (fed and fasted) (Table 2). The model that best fit the effect of dietary Phe on F^13^CO_2_ was the broken-line linear model, both when F^13^CO_2_ was expressed as a function of total BW or LSTM. A breakpoint for the mean Phe requirement with excess of Tyr, when F^13^CO_2_ as a function of total BW (mmol·kg^−1^·h^−1^) was used as the outcome of interest, was identified at 0.32% (34.03 mg·kg^−1^ BW) with an upper 95% confidence limit (**CL**) of 0.59% (62.74 mg·kg^−1^ BW) on a DM basis (Figure 1 and Table 3), on an energy density of 4,200 kcal of metabolizable energy/kg DM calculated using the modified Atwater factors. When expressed on energy density of 4,000 kcal ME/kg DM, the CL would equate to 0.56% on a DM basis. When F^13^CO_2_ as a function of LSTM (mmol·kg-LSTM^−1^·h^−1^) was used as the outcome of interest, a breakpoint for the mean Phe requirement was found at 0.31% (25.48 mg·kg^−1^ LSTM) with an upper 95% CL of 0.58% (47.66 mg·kg^−1^ LSTM) on a DM basis (Figure 1), on an energy density of 4,200 kcal of metabolizable energy/kg DM calculated using the modified Atwater factors. When expressed on energy density of 4,000 kcal ME/kg DM, the CL would be 0.55% on a DM basis.

**Table 2.** Body weight, energy expenditure, respiratory quotient, and VCO_2_/VO_2_ for cats fed diets containing graded levels of Phe (study 1)

Parameters^1^	Dietary phenylalanine, % DM									
	0.29	0.34	0.39	0.44	0.54	0.64	0.74	0.84	Pooled SEM^2^	*P* value^3^
	*n* = 4	*n* = 4	*n* = 4	*n* = 4	*n* = 4	*n* = 4	*n* = 4	*n* = 4		
BW, kg	5.37	5.36	5.37	5.38	5.38	5.34	5.34	5.36	0.444	0.69
REE, kcal/kg BW	26.75	26.26	29.54	29.46	28.06	29.47	30.52	27.23	2.484	0.73
REE, kcal/kg BW^0.67^	46.12	45.34	51.13	51.19	48.46	50.92	53.03	47.01	3.917	0.73
FEE, kcal/kg BW	32.15	36.53	35.97	37.08	38.95	38.73	36.07	39.40	3.706	0.41
FEE, kcal/kg BW^0.67^	55.22	62.89	62.09	64.61	66.42	65.06	62.05	68.16	5.369	0.52
Fasted RQ	0.74	0.76	0.76	0.75	0.74	0.76	0.75	0.76	0.013	0.76
Fed RQ	0.83	0.83	0.83	0.83	0.82	0.83	0.83	0.83	0.007	0.99
Fed VCO_2_, mL/min	21.26	22.96	22.41	23.39	23.11	23.11	23.89	24.78	1.413	0.42
Fed VO_2_, mL/min	25.48	27.37	27.07	28.11	28.31	28.06	28.75	29.82	1.720	0.40

^1^BW = body weight, FEE = fed-state energy expenditure, REE = resting energy expenditure, RQ = respiratory quotient, VCO_2_ = volume of CO_2_, VO_2_ = volume of O_2_.

^2^Standard error of the mean.

^3^Significantly different when *P* ≤ 0.05 and a tendency declared when 0.05 < *P* ≤ 0.10.

**Table 3.** Dietary Phenylalanine requirement, in the presence of excess of Tyrosine, for adult cats at maintenance as recommended by AAFCO, FEDIAF, NRC, and estimated in the present study

Units	AAFCO^1^	FEDIAF^2^	NRC^3^ AI^4^	NRC RA	Present Study	
					MR	CL
g/100 g DM^5^	0.42	0.53/0.40	0.40	0.40	0.31	0.56
g/Mcal ME	1.05	1.33/1.00	1.00	1.00	0.76	1.33
mg/kg BW					34.03	62.74
						(56.89)^6^

^1^Association of American Feed Control Officials Manual. 2023.

^2^European Pet Food Industry Federation (2023) Nutritional guidelines for complete and complementary pet food for cats and dogs. Values depend on maintenance energy requirements 75 kcal/kg^0.67^ or 100 kcal/kg^0.67^, respectively.

^3^Nutrient requirements of dogs and cats; National Research Council, 2006.

^4^AI = adequate intake, RA = Recommended allowance, MR = Minimal requirement, CL = upper 95% confidence limit.

^5^Values for g 100 g DM^−1^ are determined assuming a dietary energy density of 4,000 kcal ME kg^−1^.

^6^Values in parentheses represent NRC recommendation for Phe requirement for adult cats at maintenance converted from mg·kg^−1^ BW^0.67^ to mg·kg^−1^ BW using the average BW of cats in the present study.

## Study 2

### Study 2a: measurement of food intake

Mean intake rate (g min^−1^), time to finish (min), orts (g), and intake (% eaten) are reported in Table 4. The mean intake rate did not differ between TRT and CTRL (*P* > 0.05) but tended (*P* < 0.10) to be slower for cats on CTRL compared to cats on TRT. There was no treatment-by-day interaction effect (*P* > 0.05) for intake rate. However, the intake rate was slower on day 2 when compared to day 1 (*P* < 0.05), but intake rates for days 1 and 2 were similar (*P* > 0.05) to days 3 and 4. No effects of treatment, day, and their interaction were observed for time to finish, orts (g), and intake (% eaten) (*P* > 0.05).

**Table 4.** Mean daily food intake, time to finish, orts and intake (% eaten) for all cats (*n* = 12) when evaluating the effects of dietary Phe (44 mg kg^−1^ BW) on gastric empyting rate (study 2a)

Parameter	Treatment^1^			Day					*P* value^2^		
	CTRL	TRT	Pooled SEM	1	2	3	4	Pooled SEM	Treatment	Day	Treatment*day
Intake Rate, g min^−1^	5.67	6.07	0.518	6.27^a^	5.62^b^	5.64^ab^	5.95^ab^	0.529	0.08	0.04	0.66
Time to finish, min	14.11	13.21	1.959	13.00	13.83	13.85	13.97	1.969	0.30	0.61	0.16
Orts, g	0.44	0.57	0.484	0.45	0.23	0.35	0.99	0.476	0.80	0.23	0.99
Intake, % eaten	99.36	99.27	0.618	99.41	99.71	99.55	98.59	0.620	0.89	0.20	0.99

^1^CTRL = control; TRT = treatment; SEM = standard error of the mean.

^2^Significantly different when *P* ≤ 0.05 and a tendency declared when 0.05 < *P* ≤ 0.10.

^a,b^Values in a row with different superscripts are different within Day (*P* ≤ 0.05).

### Study 2b: GE rate and macronutrient metabolism

The enrichment of ^13^CO_2_ was successfully captured in all breath samples when using a dose of 5 mg·kg^−1^ BW of [1-^13^C]-octanoic acid. Baseline ^13^CO_2_ enrichment was defined as the ^13^CO_2_ enrichment of the first postprandial breath sample. A return to baseline ^13^CO_2_ enrichment was defined as an 80% recovery from peak ^13^CO_2_ production. In the pilot study, all cats returned to baseline enrichment by 12 h following meal consumption. When evaluating the frequency of breath samples required to capture total GE and time to peak ^13^CO_2_ production, reducing sample collection from every 20 min to every 40 min for the first 2 h of breath collection and from every 40 min to every 80 min for the following 10 h of collection produced similar estimates of total GE (AUC) (118.30 vs. 118.76; *P* > 0.05). Even though all cats returned to baseline (defined as an 80% recovery from peak ^13^CO_2_ production) in the pilot trial, only five out of twelve returned to baseline enrichment in study 2b, and thus, 7 out of 12 cats were removed from statistical analysis.

Total GE as assessed by the AUC of F^13^CO_2_ (mmol min^−1^) plotted against time (min) and time to peak ^13^CO_2_ production (min) are reported in Table 5. Total GE did not differ between TRT or CTRL (*P* > 0.05). Time to peak ^13^CO_2_ production (min), signifying peak GE did not differ between TRT and CTRL (*P* > 0.05) but tended (0.05 < *P* ≤ 0.10) to be slower for cats on TRT than cats on CTRL. In Table 6, data for mean fasted, fed, and three postprandial and total postprandial RQ values are presented for all 12 cats. No differences were detected in RQ values for cats on TRT compared to CTRL for fasted (−60 to 0 min) and all postprandial (120 to 480 min, 480 to 840 min, 840 to 1,200 min, and 120 to 1,200 min) measurements (*P* > 0.05). However, in the fed state (0 to 120 min), cats on TRT had a higher RQ value than cats on CTRL (*P* < 0.05). No differences (*P* > 0.05) for the effect of treatment were detected for EE when based on BW (Table 6). When evaluating the effect of treatment for EE based on LSTM, no differences (*P* > 0.05) were detected, but EE for cats on CTRL during 840 to 1,200 min of postprandial data collection tended (*P* < 0.10) to be lower than cats on TRT.

**Table 5.** Mean production of ^13^CO_2_ calculated by AUC produced from ^13^CO_2_ expired (mmol min^−1^) plotted against time and time to peak for cats that returned to baseline enrichment (*n* = 5) when evaluating the effects of dietary Phe (44 mg kg^−1^ BW) on GE rate when using the ^13^C-OABT (study 2b)

GE parameter	Treatment^1^			*P* value^2^
	CTRL	TRT	Pooled SEM	
AUC, mmol min^−1^ min	102	97.0	18.1	0.81
Time to peak ^13^CO_2_ production, min	260	468	65.8	0.06

^1^CTRL = control; TRT = treatment; SEM = standard error of the mean.

^2^Significantly different when *P* ≤ 0.05 and a tendency declared when 0.05 < *P* ≤ 0.10.

**Table 6.** Mean fasted, fed, and postprandial EE and RQ for all cats (*n* = 12) when evaluating GE rates using ^13^C-OABT. Cats were fed 50% of their daily ration at time 15 min (study 2b)

Calorimetry parameter^1^	Treatment^2^			
	CTRL	TRT	Pooled SEM	*P* value^2^
Fasted EE, kcal kg^−1^ BW.d^−1^ (−60 to 0 min)	37.12	40.64	2.799	0.31
Fed EE, kcal kg^−1^ BW d^−1^ (0 to 120 min)	38.88	42.04	2.991	0.26
Postprandial EE 1, kcal kg^−1^ BW d^−1^ (120 to 480 min)	38.08	39.09	2.010	0.63
Postprandial EE 2, kcal kg^−1^ BW d^−1^ (480 to 840 min)	40.73	42.30	2.102	0.37
Postprandial EE 3, kcal kg^−1^ BW d^−1^ (840 to 1,200 min)	39.02	42.24	2.109	0.16
Postprandial EE total, kcal kg^−1^ BW d^−1^ (120 to 1,200 min)	39.26	40.77	1.922	0.40
Fasted EE, kcal kg-LSTM^−1^ d^−1^ (−60 to 0 min)	47.20	49.84	2.832	0.36
Fed EE, kcal kg-LSTM^−1^ d^−1^ (0 to 120 min)	49.70	51.65	3.191	0.38
Postprandial EE 1, kcal kg-LSTM^−1^ d^−1^ (120 to 480 min)	48.85	47.84	2.067	0.46
Postprandial EE 2, kcal kg-LSTM^−1^ d^−1^ (480 to 840 min)	52.04	51.18	2.367	0.41
Postprandial EE 3, kcal kg-LSTM^−1^ d^−1^ (840 to 1,200 min)	49.69	51.58	1.961	0.08
Postprandial EE total, kcal kg-LSTM^−1^ d^−1^ (120 to 1,200 min)	50.18	48.69	2.343	0.30
Fasted RQ (−60 to 0 min)	0.73	0.74	0.006	0.22
Fed RQ (0 to 120 min)	0.71^b^	0.73^a^	0.004	<0.01
Postprandial 1 RQ (120 to 480 min)	0.78	0.77	0.004	0.26
Postprandial 2 RQ (480 to 840 min)	0.79	0.79	0.005	0.85
Postprandial 3 RQ (840 to 1,200 min)	0.76	0.76	0.006	0.86
Postprandial RQ total (120 to 1,200 min)	0.77	0.77	0.004	0.55

^1^BW = body weight; EE = energy expenditure; LSTM = lean soft tissue mass; RQ = respiratory quotient.

^2^CTRL = control; TRT = treatment; SEM = standard error of the mean.

^3^Significantly different when *P* ≤ 0.05 and a tendency declared when 0.05 < *P* ≤ 0.10.

^a,b^Values in a row with different superscripts are different (*P* ≤ 0.05).

## Discussion

To our knowledge, this is the first study to empirically determine the minimum Phe requirement, with excess of Tyr, in adult cats. To account for variability within the population, we compared the estimated 95% CL with the level for AI recommended in the NRC (2006). Our 95% CL was ~47% higher than the AI put forth by the NRC (2006) and ~40% higher than the Phe requirements suggested by the Association of American Feed Control Officials (AAFCO, 2023) for adult cats at maintenance. There were no changes in calorimetry responses across all dietary Phe levels evaluated, signifying that differing levels of Phe did not affect energy or macronutrient metabolism and that all changes seen in F^13^CO_2_ production were a result of differences in the dietary intake of Phe. These results are similar to those observed in carbon oxidation in dogs (Mansilla et al., 2018, 2020a,b; Templeman et al., 2019; Sutherland et al., 2020), where EE and RQ also remained unchanged across different dietary levels of the test AA, and the requirement of the AA in question was found to be higher than estimates provided by growth and nitrogen balance research. Both growth and nitrogen balance methodologies lack the sensitivity required for the stability of body protein pools in adult animals.

The findings of study 1 suggest that the AI and RA for Phe recommended by the NRC (2006) and by AAFCO (2023) for commercial feline diets are underestimated. Only neutered male cats were used in the present study, so future investigation into Phe requirements in different neuter statuses and sex is warranted. It is important to note that while Phe concentrations in the basal diet were provided from intact protein sources, graded levels of Phe across experimental diets were achieved with crystalline Phe, assumed to have 100% bioavailability. As such, the estimate of the requirement determined in our study cannot be classified as a minimal (physiological) requirement and would fall somewhere between the MR and RA of the NRC (2006). Commercial cat foods utilize intact proteins to fulfill AA requirements and can be highly processed, which can reduce AA bioavailability (van Rooijen et al., 2013). Thus, recommendations for commercial pet food should be higher than the current determination.

As ^13^C-Phe is used as the main indicator AA in carbon oxidation studies, the minimum requirement for Phe must be defined for future IAAO studies to ensure Phe is not limiting protein synthesis and all responses are due to the intake of the test AA. Of the indispensable AA for adult cats, the NRC has only defined MR for Tau, Lys, Met, and total sulfur AA (NRC, 2006). We recently applied the IAAO technique to determine the minimum methionine requirement in adult cats (Pezzali et al., 2024) and also found higher estimates than those proposed by the NRC (2006) and AAFCO (2023). While the Met requirement study (Pezzali et al., 2024) was conducted before the current study, the estimates of the Phe requirement in the present study indicate that Pezzali et al., (2024) provided sufficient amounts of Phe when estimating the methionine requirement. The protocol utilized in the current study was successful due to modifications on the length of the adaption period to the basal diet (7 d) and experimental diets (1 d) from the suggestions proposed by Pezzali et al., (2024) as difficulties were faced in achieving the ideal sample size with longer adaptation periods. Thus, future IAAO studies should follow this adapted protocol.

While we were successful at applying the DAAO technique in adult cats, it is important to emphasize that the breakpoint was identified between diets 1 and 2, making it difficult to determine a clear curve response as a minimum of three points are necessary to satisfactory define a line (Pencharz and Ball, 2003). Thus, a basal diet with a dietary Phe concentration lower than 0.29% DM would be ideal to define a breakpoint with three dietary levels below it. However, developing a palatable semi-synthetic diet lower in Phe presents challenges. Cats have a preferential diet selection for protein (Hewson-Hughes et al., 2011), thus, diets both low in protein content and formulated using crystalline AA, as opposed to intact animal-based proteins, are not very palatable and may result in refusal over time. Our approach represented the best compromise between keeping Phe low and ensuring the cats’ dietary acceptance. Additionally, it is important to point out that the high SEM value for the breakpoint estimate (0.32 ± 0.085%) has had an influence on the wide range observed for the 95% CL. Even though the between-subject variance in amino acid requirements is significantly high, and we attempt to account for it using a crossover experimental design, a larger sample size may be necessary for cats to enhance the accuracy of the breakpoint estimate.

The work presented in study 2, first sought to validate the use of the ^13^C-OABT in cats, to then be utilized for the evaluation of the effects of dietary Phe supplementation on GE. Feline obesity affects upwards of 60% of the global cat population (Cave et al., 2012; Courcier et al., 2012; Wall et al., 2019). With multiple comorbidities resulting from obesity, we investigated the potential use of supplemental Phe for the prevention, rather than treatment of feline obesity, through its potential satiety-inducing effects. The pilot data indicated that a dose of 5 mg·kg^−1^ BW of ^13^C-Octanoic acid was successful at capturing GE in cats, with a return to baseline ^13^CO_2_ enrichment within 12 h of meal consumption. However, under the same sampling protocol as the pilot trial, in study 2a, only 5 of 12 cats returned to baseline. Of the four cats used in the pilot trial, two were used in study 2a, and only one of the cats had a successful return to baseline. While stress can affect GE rates (Gué et al., 1989) this is unlikely in our studies, as the cats had been previously acclimated to the environment, people, and calorimetry chamber system (Gooding et al., 2012) and exhibited no signs of stress throughout both the pilot study and the GE study. This suggests the potential for GE rates to be influenced by day-to-day variation. Future studies should evaluate the recovery of ^13^CO_2_ from the administered dose, as this investigation may elucidate the significant variability observed across all cats, and the reasons behind the return to baseline enrichment in only 5 out of the 12 cats.

While Phe supplementation at 44 mg·kg^−1^ BW did not influence food intake, macronutrient metabolism, or total GE, there was a tendency for Phe to delay time to peak GE suggesting that Phe may reduce the rate of GE. Cats on Phe tended to have peak emptying at roughly 3.7 h later than cats on Ala, signifying that Phe may play a role in regulating peak GE. Nonetheless, despite the limited sample size, the results reveal a trend, suggesting that further exploration is warranted to investigate whether Phe truly has the potential to extend the time to peak GE. Future studies should also consider a different basal diet, as it has been speculated that CCK secretion may be adapted to diet composition; therefore, dietary protein has been suggested as secretagogues of CCK in cats (Backus et al., 1995). Backus et al. (1995) found that intact protein and individual AAs elevate plasma CCK in cats. Thus, it may be warranted to use diets lower in protein in future studies to confirm that a delay in GE is in response to Phe supplementation stimulating CCK release and not a result of dietary protein intake.

The doses of Phe used in studies 2a and 2b were determined based on previous research by Backus and Rogers (1996), where the provision of 44 mg·kg^−1^ BW of Phe elevated plasma CCK in cats. However, the effects of increased plasma CCK on the satiety response were not evaluated, so it is difficult to conclude if this dose is sufficient to elicit an adequate elevation in CCK concentrations to further cause a delay in GE and thereby reduce feed intake through the promotion of satiation. It is important to note that studies 2a and 2b did not evaluate plasma CCK concentrations of the cats following Phe consumption, as the cats used in our study required sedation to safely collect blood, and blood collection should be done in the fed state when evaluating CCK; thus, future research should measure plasma CCK levels following Phe consumption to determine if Phe truly elevates plasma CCK under these conditions, as well as determine if CCK follows GE patterns in cats. It is important to note that AA seems to impact GE by osmotic mechanism, with concentrations <80 mM being below the predicted threshold range for duodenal osmoreceptors (Stephens et al., 1975). Phe and Tyr differ from the other AA as they are postulated to have these effects when at concentrations <80 mM, however, the dose of Phe used was ~10 mM·kg^−1^ BW, which may have been too low to see significant effects. It may also be worth investigating additional doses of Phe to evaluate the effects on a satiety response and food intake. Furthermore, while CCK is one of many hormones involved in satiety induction, it is possible that stimulation of multiple hormones together may be necessary to see a reduction in food intake, GE, and macronutrient metabolism in the domestic cat.

## Conclusion

The breakpoint for the mean Phe requirement with the presence of excess Tyr, was identified at 0.32% (34.03 mg·kg^−1^ BW) on a DM basis on an energy density of 4,200 kcal of metabolizable energy/kg DM calculated using the modified Atwater factors. The upper 95% CL (0.59% DM) estimated in this study for Phe in the adult cat is higher than the recommendations made by the NRC (0.40% DM) and AAFCO (0.42% DM). The ^13^C-OABT combined with indirect calorimetry chambers to collect breath samples can be used to evaluate GE in adult cats. A bolus dose of Phe did not influence food intake, GE, or macronutrient metabolism as hypothesized. While there was a trend for cats on Phe to have delayed times to peak GE, our results should be interpreted with caution, and future studies should investigate the effects of alternative doses and explore the length of supplementation of Phe on food intake, GE, and macronutrient metabolism together with satiety hormones.

## Abbreviations

^13^C-OABT: ^13^C-octanoic acid breath test
AA: amino acid
AAFCO: American Association of Feed Control Officials
AI: adequate intake
APE: atom percent excess
AUC: area under the curve
BW: body weight
CCK: cholecystokinin
CL: confidence limit
DAAO: direct amino acid oxidation
DM: dry matter
EE: energy expenditure
F^13^CO_2_: flux of ^13^CO_2_
FEDIAF: European Pet Food Industry Federation
FEE: fed-state energy expenditure
GE: gastric emptying
IAAO: indicator amino acid oxidation
LSM: least square means
LSTM: lean soft tissue mass
MR: minimal requirement
NRC: National Research Council
RA: recommended allowance
REE: resting energy expenditure
RQ: respiratory quotient
VCO_2_: volume of CO_2_
VO_2_: volume of O_2_

## References

[CIT0001] AAFCO. 2023. AAFCO Official Publication. Saint Paul, Minnesota: AAFCO, Inc.

[CIT0002] Anderson, P. A., D. H. Baker, P. A. Sherry, and J. E. Corbin. 1980. Histidine, phenylalanine-tyrosine and tryptophan requirements for growth of the young kitten. J. Anim. Sci. 50:479–483. doi:10.2527/jas1980.503479x 7364684

[CIT0003] Anderson, P. J. B., Q. R. Rogers, and J. G. Morris. 2002. Cats require more dietary phenylalanine or tyrosine for melanin deposition in hair than for maximal growth. J. Nutr. 132:2037–2042. doi:10.1093/jn/132.7.2037 12097689

[CIT0004] Backus, R. C., and Q. Rogers. 1996. Phenylalanine is a physiological stimulus of endocrine cholecystokinin (CCK) secretion in cats. FASEB J. 10

[CIT0005] Backus, R. C., G. L. Rosenquist, Q. R. Rogers, J. Calam, and J. G. Morris. 1995. Elevation of plasma cholecystokinin (CCK) immunoreactivity by fat, protein, and amino acids in the cat, a carnivore. Regul. Pept. 57:123–131. doi:10.1016/0167-0115(95)00027-9 7659788

[CIT0006] Cave, N. J., F. J. Allan, S. L. Schokkenbroek, C. A. M. Metekohy, and D. U. Pfeiffer. 2012. A cross-sectional study to compare changes in the prevalence and risk factors for feline obesity between 1993 and 2007 in New Zealand. Prev. Vet. Med. 107:121–133. doi:10.1016/j.prevetmed.2012.05.006 22703979

[CIT0007] Courcier, E. A., D. J. Mellor, E. Pendlebury, C. Evans, and P. S. Yam. 2012. An investigation into the epidemiology of feline obesity in Great Britain: results of a cross-sectional study of 47 companion animal practises. Vet. Rec. 171:560–560. doi:10.1136/vr.100953 23081976

[CIT0008] Daly, K., M. Al-Rammahi, A. Moran, M. Marcello, Y. Ninomiya, and S. P. Shirazi-Beechey. 2013. Sensing of amino acids by the gut-expressed taste receptor T1R1-T1R3 stimulates CCK secretion. Am. J. Physiol. Gastrointest. Liver Physiol. 304:G271–G282. doi:10.1152/ajpgi.00074.2012 23203156 PMC3566511

[CIT0009] Debas, H. T., O. Farooq, and M. I. Grossman. 1975. Inhibition of gastric emptying is a physiological action of cholecystokinin. Gastroenterology. 68:1211–1217. doi:10.1016/S0016-5085(75)80236-8 1126597

[CIT0010] Dickinson, P. J., P. J. Anderson, D. C. Williams, H. C. Powell, D. G. Shelton, J. G. Morris, and R. A. LeCouteur. 2004. Assessment of the neurologic effects of dietary deficiencies of phenylalanine and tyrosine in cats. Am. J. Vet. Res. 65:671–680. doi:10.2460/ajvr.2004.65.671 15141890

[CIT0011] Di Lorenzo, C., C. M. Williams, F. Hajnal, and J. E. Valenzuela. 1988. Pectin delays gastric emptying and increases satiety in obese subjects. Gastroenterology. 95:1211–1215. doi:10.1016/0016-5085(88)90352-6 3169489

[CIT0012] Dratman, M. B. 1974. On the mechanism of action of thyroxin, an amino acid analog of tyrosine. J. Theor. Biol. 46:255–270. doi:10.1016/0022-5193(74)90151-9 4152982

[CIT0013] Elango, R., R. O. Ball, and P. B. Pencharz. 2008. Individual amino acid requirements in humans: an update. Curr. Opin Clin. Nutr. Metab. Care. 11:34–39. doi:10.1097/MCO.0b013e3282f2a5a4 18090656

[CIT0014] Elango, R., R. O. Ball, and P. B. Pencharz. 2012. Recent advances in determining protein and amino acid requirements in humans. Br. J. Nutr. 108(Supp 2):S22–S30. doi:10.1017/S0007114512002504 23107531

[CIT0015] Enç, F. Y., N. I˙meryüz, L. Akin, T. Turoğlu, F. Dede, G. Haklar, N. Tekeşi˙n, N. Beki˙roğlu, B. Yeğen, J. F. Rehfeld, et al. 2001. Inhibition of gastric emptying by acarbose is correlated with GLP-1 response and accompanied by CCK release. Am. J. Physiol. Gastrointest. Liver. Physiol. l281:G752–G763. doi:10.1152/ajpgi.2001.281.3.G752 11518688

[CIT0016] Gibbs, J., R. C. Young, and G. P. Smith. 1973. Cholecystokinin decreases food intake in rats. J. Comp. Physiol. Psychol. 84:488–495. doi:10.1037/h0034870 4745816

[CIT0017] Gooding, M. A., I. J. H. Duncan, J. L. Atkinson, and A. K. Shoveller. 2012. Development and validation of a behavioral acclimation protocol for cats to respiration chambers used for indirect calorimetry studies. J. Appl. Anim. Welf. Sci. 15:144–162. doi:10.1080/10888705.2012.658332 22458875

[CIT0018] Gué, M., T. Peeters, I. Depoortere, G. Vantrappen, and L. Buéno. 1989. Stress-induced changes in gastric emptying, postprandial motility, and plasma gut hormone levels in dogs. Gastroenterology. 97:1101–1107. doi:10.1016/0016-5085(89)91678-8 2571543

[CIT0019] Hewson-Hughes, A. K., V. L. Miller, S. R. Hall, S. J. Simpson, and D. Raubenheimer. 2011. Geometric analysis of macronutrient selection in the adult domestic cat, Felis catus. J. Exp. Biol. 214:1039–1051. doi:10.1242/jeb.049429 21346132

[CIT0020] Hilton, M. A., M. L. Fonda, and F. K. Hilton. 1998. The effect of tyrosine-deficient total parenteral nutrition on the synthesis of dihydroxyphenylalanine in neural tissue and the activities of tyrosine and branched-chain aminotransferases. Metab. Clin. Exp. 47:168–176. doi:10.1016/s0026-0495(98)90215-3 9472965

[CIT0021] Hira, T., S. Nakajima, Y. Eto, and H. Hara. 2008. Calcium-sensing receptor mediates phenylalanine-induced cholecystokinin secretion in enteroendocrine STC-1 cells. FEBS J. 275:4620–4626. doi:10.1111/j.1742-4658.2008.06604.x 18691347

[CIT0055] Hogan, K., N. Genova, J. R. Templeman, A. Verbrugghe, and A. K. Shoveller. 2022. Introduction of adult cats to indirect calorimetry respiration chambers causes increased energy expenditure and respiratory quotient that decrease following acclimation. *A. J. Vet. Re*. 83(3):264–269. doi:10.2460/ajvr.20.10.0185 34986115

[CIT0022] Hopman, W. P., J. B. Jansen, and C. B. Lamers. 1985. Comparative study of the effects of equal amounts of fat, protein, and starch on plasma cholecystokinin in man. Scand. J. Gastroenterol. 20:843–847. doi:10.3109/00365528509088832 4048835

[CIT0023] Humayun, M. A., R. Elango, R. O. Ball, and P. B. Pencharz. 2007. Reevaluation of the protein requirement in young men with the indicator amino acid oxidation technique. Am. J. Clin. Nutr. 86:995–1002. doi:10.1093/ajcn/86.4.995 17921376

[CIT0024] Hunt, J. N. 1980. A possible relation between the regulation of gastric emptying and food intake. Am. J. Physiol. 239:G1–G4. doi:10.1152/ajpgi.1980.239.1.G1 7395999

[CIT0025] Liddle, R. A., E. T. Morita, C. K. Conrad, and J. A. Williams. 1986. Regulation of gastric emptying in humans by cholecystokinin. J. Clin. Invest. 77:992–996. doi:10.1172/JCI112401 3949984 PMC423501

[CIT0026] Mansilla, W. D., A. Gorman, L. Fortener, and A. K. Shoveller. 2018. Dietary phenylalanine requirements are similar in small, medium, and large breed adult dogs using the direct amino acid oxidation technique. J. Anim. Sci. 96:3112–3120. doi:10.1093/jas/sky208 29846616 PMC6095264

[CIT0027] Mansilla, W. D., L. Fortener, J. R. Templeman, and A. K. Shoveller. 2020a. Adult dogs of different breed sizes have similar threonine requirements as determined by the indicator amino acid oxidation technique. J. Anim. Sci. 98:skaa066. doi:10.1093/JAS/SKAA066 32108874 PMC7085255

[CIT0028] Mansilla, W. D., J. R. Templeman, L. Fortener, and A. K. Shoveller. 2020b. Minimum dietary methionine requirements in Miniature Dachshund, Beagle, and Labrador Retriever adult dogs using the indicator amino acid oxidation technique. J. Anim. Sci. 98:skaa324 doi:10.1093/jas/skaa324 33011778 PMC7751151

[CIT0029] Marathe, C. S., C. K. Rayner, K. L. Jones, and M. Horowitz. 2013. Relationships between gastric emptying, postprandial glycemia, and incretin hormones. Diabetes Care 36:1396–1405. doi:10.2337/dc12-1609 23613599 PMC3631884

[CIT0030] NRC. 2006. Nutrient requirements of dogs and cats. Washington, DC: Natl. Acad. Press

[CIT0031] Peachey, S. E., J. M. Dawson, and E. J. Harper. 2000. Gastrointestinal transit times in young and old cats. Comp. Biochem. Physiol. A: Mol. Integr. Physiol. 126:85–90. doi:10.1016/s1095-6433(00)00189-6 10908855

[CIT0032] Pencharz, P. B., and R. O. Ball. 2003. Different approaches to define individual amino acid requirements. Annu. Rev. Nutr. 23:101–116. doi:10.1146/annurev.nutr.23.011702.073247 12626690

[CIT0033] Pezzali, J. G., A. Bullerwell, K. Dancy, T. J. DeVries, and A. K. Shoveller. 2023a. The development of a semi-synthetic diet deficient in Methionine for adult cats for controlled feline nutrition studies: effects on acceptability, preference and behavior responses. J. Anim. Sci. 101:skac392. doi:10.1093/jas/skac392 36440575 PMC9838793

[CIT0034] Pezzali, J. G., M. Rafii, G. Courtney-Martin, and A. K. Shoveller. 2023b. Applying the indicator amino acid oxidation technique in the domestic cat: results of a pilot study and development of a non-steady state prediction model. J. Anim. Sci. 101:skac390. doi:10.1093/jas/skac390 36434784 PMC10007698

[CIT0035] Pezzali, J. G., J. G. Lambie, S. M. Phillips, and A. K. Shoveller. 2023c. Determination of a steady state isotope dilution protocol for carbon oxidation studies in the domestic cat. J. Nutr. Sci. 12:e62. doi:10.1017/jns.2023.44 37313346 PMC10260335

[CIT0036] Pezzali, J. G., J. G. Lambie, A. Verbrugghe, and A. K. Shoveller. 2024. Minimum methionine requirement in adult cats as determined by indicator amino acid oxidation. J. Anim. Sci. 102:skad411. doi:10.1093/jas/skad411 38092464 PMC10768993

[CIT0037] Raper, H. S., and A. Wormall. 1923. The tyrosinase-tyrosine reaction. Biochem. J. 17:454–469. doi:10.1042/bj0170454 16743237 PMC1263908

[CIT0038] Raybould, H. E., and Y. Tache. 1988. Cholecystokinin inhibits gastric motility and emptying via a capsaicin-sensitive vagal pathway in rats. Am J Physiol 255:G242–G246. doi:10.1152/ajpgi.1988.255.2.G242 3136661

[CIT0039] Richards, T. L., A. Rankovic, J. P. Cant, A. K. Shoveller, J. L. Adolphe, D. Ramdath, and A. Verbrugghe. 2021. Effect of total starch and resistant starch in commercial extruded dog foods on gastric emptying in non-racing sled dogs. Animals. 11(10):2928. doi:10.20944/preprints202107.0689.v1 34679949 PMC8532653

[CIT0040] Rogers, Q. R., and J. G. Morris. 1979. Essentiality of amino acids for the growing kitten. J. Nutr. 109:718–723. doi:10.1093/jn/109.4.718 430271

[CIT0041] Shiman, R., and D. W. Gray. 1998. Formation and fate of tyrosine Intracellular partitioning of newly synthesized tyrosine in mammalian liver. J. Biol. Chem. 273:34760–34769. doi:10.1074/jbc.273.52.34760 9857000

[CIT0042] Shoveller, A. K., J. J. Danelon, J. L. Atkinson, G. M. Davenport, R. O. Ball, and P. B. Pencharz. 2017. Calibration and validation of a carbon oxidation system and determination of the bicarbonate retention factor and the dietary phenylalanine requirement, in the presence of excess tyrosine, of adult, female, mixed-breed dogs. J. Anim. Sci. 95:2917–2927. doi:10.2527/jas.2017.1535 28727110

[CIT0043] Stephens, J. R., R. F. Woolson, and A. R. Cooke. 1975. Effects of essential and nonessential amino acids on gastric emptying in the dog. Gastroenterology. 69:920–927. 1175886

[CIT0044] Sutherland, K. A. K., W. D. Mansilla, L. Fortener, and A. K. Shoveller. 2020. Lysine requirements in small, medium, and large breed adult dogs using the indicator amino acid oxidation technique. Transl. Anim. Sci. 4:txaa082. doi:10.1093/tas/txaa082 32734145 PMC7381836

[CIT0045] Templeman, J. R., W. D. Mansilla, L. Fortener, and A. K. Shoveller. 2019. Tryptophan requirements in small, medium, and large breed adult dogs using the indicator amino acid oxidation technique. J. Anim. Sci. 97:3274–3285. doi:10.1093/jas/skz142 31363781 PMC6667247

[CIT0046] van Rooijen, C., G. Bosch, A. F. van der Poel, P. A. Wierenga, L. Alexander, and W. H. Hendriks. 2013. The Maillard reaction and pet food processing: effects on nutritive value and pet health. Nutr. Res. Rev. 26:130–148. doi:10.1017/S0954422413000103 23916186

[CIT0047] Wall, M., N. J. Cave, and E. Vallee. 2019. Owner and cat-related risk factors for feline overweight or obesity. Front. Vet. Sci. 6:266. doi:10.3389/fvets.2019.00266 31482097 PMC6709657

[CIT0048] Wang, Y., R. Chandra, L. A. Samsa, B. Gooch, B. E. Fee, J. M. Cook, S. R. Vigna, A. O. Grant, and R. A. Liddle. 2011. Amino acids stimulate cholecystokinin release through the Ca2+-sensing receptor. Am. J. Physiol. Gastrointest. Liver Physiol. 300:G528–G537. doi:10.1152/ajpgi.00387.2010 21183662 PMC3074989

[CIT0049] Watson, A., J. Wayman, R. Kelley, A. Feugier, and V. Biourge. 2018. Increased dietary intake of tyrosine upregulates melanin deposition in the hair of adult black-coated dogs. Anim. Nutr. 4:422–428. doi:10.1016/j.aninu.2018.02.001 30564763 PMC6286625

[CIT0050] Weir, J. B. 1949. New methods for calculating metabolic rate with special reference to protein metabolism. J. Physiol. 109:1–9. doi:10.1113/jphysiol.1949.sp004363 15394301 PMC1392602

[CIT0051] Williams, J. M., J. G. Morris, and Q. R. Rogers. 1987. Phenylalanine requirement of kittens and the sparing effect of tyrosine. J. Nutr. 117:1102–1107. doi:10.1093/jn/117.6.1102 3598720

[CIT0052] Yamagishi, T., and H. T. Debas. 1978. Cholecystokinin inhibits gastric emptying by acting on both proximal stomach and pylorus. Am. J. Physiol. 234:E375–E378. doi:10.1152/ajpendo.1978.234.4.E375 645853

[CIT0053] Yu, S., Q. R. Rogers, and J. G. Morris. 2001. Effect of low levels of dietary tyrosine on the hair colour of cats. J. Small Anim. Pract. 42:176–180. doi:10.1111/j.1748-5827.2001.tb01798.x 11327664

[CIT0054] Zello, G. A., L. J. Wykes, R. O. Ball, and P. B. Pencharz. 1995. Recent advances in methods of assessing dietary amino acid requirements for adult humans. J. Nutr. 125:2907–2915. doi:10.1093/jn/125.12.2907 7500168

